# Towards Control of the Size, Composition and Surface Area of NiO Nanostructures by Sn Doping

**DOI:** 10.3390/nano11020444

**Published:** 2021-02-10

**Authors:** María Taeño, David Maestre, Julio Ramírez-Castellanos, Shaohui Li, Pooi See Lee, Ana Cremades

**Affiliations:** 1 Departamento de Física de Materiales, Facultad de Ciencias Físicas, Universidad Complutense de Madrid, 28040 Madrid, Spain; davidmaestre@fis.ucm.es (D.M.); cremades@fis.ucm.es (A.C.); 2 Departamento de Química Inorgánica, Facultad de Ciencias Químicas, Universidad Complutense de Madrid, 28040 Madrid, Spain; jrcastel@quim.ucm.es; 3 School of Materials Science and Engineering, Nanyang Technological University, 50 Nanyang Avenue, Singapore 639798, Singapore; lishaohui@ntu.edu.sg (S.L.); pslee@ntu.edu.sg (P.S.L.)

**Keywords:** nickel oxide, nanoparticles, nanosticks, high surface area, doping mechanisms

## Abstract

Achieving nanostructures with high surface area is one of the most challenging tasks as this metric usually plays a key role in technological applications, such as energy storage, gas sensing or photocatalysis, fields in which NiO is gaining increasing attention recently. Furthermore, the advent of modern NiO-based devices can take advantage of a deeper knowledge of the doping process in NiO, and the fabrication of p-n heterojunctions. By controlling experimental conditions such as dopant concentration, reaction time, temperature or pH, NiO morphology and doping mechanisms can be modulated. In this work, undoped and Sn doped nanoparticles and NiO/SnO_2_ nanostructures with high surface areas were obtained as a result of Sn incorporation. We demonstrate that Sn incorporation leads to the formation of nanosticks morphology, not previously observed for undoped NiO, promoting p-n heterostructures. Consequently, a surface area value around 340 m^2^/g was obtained for NiO nanoparticles with 4.7 at.% of Sn, which is nearly nine times higher than that of undoped NiO. The presence of Sn with different oxidation states and variable Ni^3+^/Ni^2+^ ratio as a function of the Sn content were also verified by XPS, suggesting a combination of two charge compensation mechanisms (electronic and ionic) for the substitution of Ni^2+^ by Sn^4+^. These results make Sn doped NiO nanostructures a potential candidate for a high number of technological applications, in which implementations can be achieved in the form of NiO–SnO_2_ p-n heterostructures.

## 1. Introduction

In the last few years, nickel oxide (NiO) has been extensively studied due to its interesting optoelectronic and magnetic properties, and its high thermal and chemical stability [1], which make it a promising candidate for numerous technological applications such as electrochromic devices [2], supercapacitors [3], photocatalyst [4,5] or gas sensors [6,7], among others. Although several publications related to Sn doped NiO nanostructures can be found in the literature, less has been done in the study of the fundamental properties of this oxide. A deep knowledge of the properties of p-type semiconductor oxides is essential in order to understand the mechanisms related to the final applications. As some examples, substitutional metal cations with different oxidation states, such as Nb^5+^, Sn^4+^, Li^+^ or Al^3+^, can lead to changes in the NiO stoichiometry and therefore can be used to tune the concentration of Ni vacancies, which usually play a key role in the applications [6,8]. It is well known that an improvement in the physical and chemical properties, and therefore in the technological applications can be achieved by using nanomaterials due to their higher porosity and huge surface-to-volume ratio [9,10]. Many efforts have been focused so far on the engineering of NiO morphology to provide nanostructures with high porosity and high surface area by doping with different elements [11,12,13]. On the other hand, SnO_2_ has been considered as a promising candidate in several technological applications, such as solar cells [14], gas sensing [15] or Li-ion batteries [16], among others. The electronic structure, optical properties or vibrational modes of this well-known n-type oxide have been deeply studied over the last few decades, making this material one of the most common dopants in NiO. Different authors have reported the influence of Sn incorporation in well-known semiconductor oxides, such as ZnO [17,18], In_2_O_3_ [19] or Ga_2_O_3_ [20], among others, confirming an improvement of the optical properties or an enhanced acetone gas sensing behaviour. However, Sn doping in NiO has attracted considerable attention due to several factors, such as its thermal and chemical stability, and the promoted variations in the NiO electrical properties. In addition, Ni^2+^ can be easily substituted by Sn^4+^ and Sn incorporation usually leads to an increased conductivity of NiO. Recent works reported on high surface area values for diverse Sn doped NiO nanostructures synthesized following different routes. Wang et al. [6] reported the growth of Sn-doped three dimensionally ordered macroporous NiO following a colloidal crystal template method, with values of surface area below 110 m^2^/g. Kim et al. [21] reported a surface area value of 87.9 m^2^/g for Sn doped NiO microspheres synthesized by spray pyrolysis and sequent heat treatment. Gao et al. [12] reported a simple hydrothermal route to the synthesis of Sn-doped NiO hierarchical nanostructures, confirming that Sn incorporation leads to a decrease in the particle size. It is well known that, among other chemical routes, hydrothermal methods usually allow for obtaining nanometric materials with high compositional and morphological control as well as nanostructures with lower dimensions, mainly due to the pressure, which plays a key role during the synthesis. Most of the works related to Sn doped NiO are focused on the effect of Sn incorporation in technological applications, such as gas sensors or photocatalyst, assuming that Sn is mainly incorporated as Sn^4+^. In addition, in those works, the Sn concentration never exceeds values around 10 at.%, not exploring the solubility limit of Sn in NiO, one aspect that motivates this study. Furthermore, Sn incorporation usually does not promote significant changes in the NiO morphology in comparison with undoped NiO. In that frame, our work can contribute with novelty and new forefront insights since the study of the solubility limit of Sn in NiO or the study of different morphologies as a function of the Sn content have been deeply discussed. In this work, undoped and Sn doped NiO nanostructures were obtained by a modified hydrothermal method. In addition, the Sn doping process and the solubility limit of Sn in NiO were explored by adding nominal atomic concentrations of Sn between 3% and 30%. Diverse complementary characterization techniques were used in the investigation of all the samples, such as X-ray diffraction (XRD), scanning and transmission electron microscopy (SEM and TEM), Raman spectroscopy, cathodoluminescence (CL), N_2_ adsorption–desorption isotherms and Brunauer–Emmett–Teller (BET) surface, and X-ray photoelectron spectroscopy (XPS).

## 2. Experimental Details

### 2.1. Synthesis of Undoped and Sn Doped NiO Nanostructures

**Figure 1:**
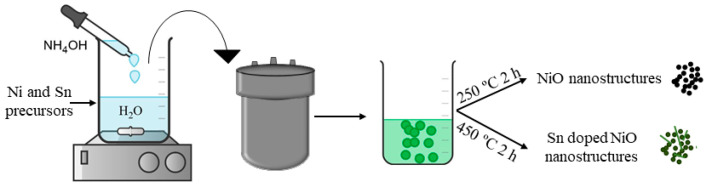
Schematic illustration of the experimental method employed to obtain undoped and Sn doped NiO.

NiO nanostructures were obtained following a soft chemical route based on a hydrothermal method followed by a thermal treatment using Ni(NO_3_)_2_·9H_2_O (Sigma-Aldrich 99.99%, Darmstadt, Germany) as precursor. The desired amount of Ni(NO_3_)_2_·9H_2_O was dissolved in deionized water under continuous stirring, then NH_4_OH was added dropwise until pH 10 was reached. The solution was transferred to a Teflon-liner autoclave and heated at 60 °C during 8 h. The final powder was collected by centrifugation, washed in deionized water and dried at 40 °C for 12 h, obtaining the Ni(OH)_2_ precursor. Thermal treatments were performed at 250 °C during 2 h to obtain NiO nanostructures. Sn doped nanostructures were obtained following a similar procedure. The desired amounts of Ni(NO_3_)_2_·9H_2_O and SnCl_2_· 2H_2_O (Sigma-Aldrich 99.99%, Darmstadt, Germany, with Sn atomic percentages of 0, 3, 6, 10, 15 and 30) were dissolved in deionized water under continuous stirring, then NH_4_OH was added dropwise until pH 10 was reached. In this case, and based on our previous work [22], the solution was heated at 120 °C during 12 h. For Sn doped NiO samples, thermal treatments were carried out at 450 °C during 2 h to obtain Sn doped NiO nanostructures. The as-obtained Sn doped NiO samples were referred to as NiO, NiO3, NiO6, NiO10, NiO15 and NiO30 based on the corresponding initial Sn atomic percentage (at.%) 0, 3, 6, 10, 15 and 30, respectively. A schematic illustration of the experimental procedure is shown in Figure 1.

### 2.2. Characterization

Structural characterization of the samples was performed by X-ray diffraction (XRD) in a Philips X’Pert Pro diffractometer (Malvern PANanalytical, UK) using Cu Kα radiation (λ = 1.54158 Å) in Bragg–Brentano configuration and by Raman spectroscopy in a Horiba Jobin–Yvon LabRaman Hr800 confocal microscope (Horiba, Kyoto, Japan) using a He–Cd laser (λ = 325 nm) as excitation source. The microstructure of the samples was analyzed by TEM and HRTEM using a JEOL JEM 3000F microscope (JEOL, Tokyo, Japan) equipped with an energy dispersive X-ray spectroscopy (EDS) detector. Compositional mappings and EDS spectra were acquired in a Leica 440 Stereoscan SEM (Leica, Wetzlar, Germany) equipped with a Bruker AXS 4010 detector. N_2_ adsorption–desorption isotherms and Brunauer–Emmett–Teller (BET) surface areas were measured in collaboration with the Nanyang Technological University, using a Tristar II 3020 instrument (Micrometrics Instrument Corporation, Singapore, Singapore) at liquid nitrogen temperature. X-ray photoelectron spectroscopy (XPS) analysis was carried out at the CNR Beamline for Advanced diCHroism (BACH) of the Elettra synchrotron facility in Trieste, Italy. Photoemission spectra were acquired with a Scienta R3000 electron energy analyzer in normal emission geometry using 650 eV photon energy with a total energy resolution of 180 meV. Finally, cathodoluminescence (CL) measurements were performed at room temperature in a Hitachi S2500 SEM (Hitachi, Tokyo, Japan) using an Oriel Cornerstone 1/4m monochromator and a Hamamatsu R928 photomultiplier.

## 3. Results and Discussion

**Figure 2:**
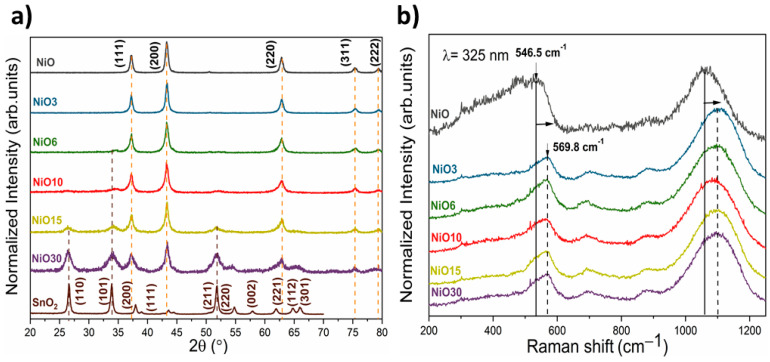
(**a**) XRD patterns from undoped and Sn doped NiO samples. XRD pattern from SnO_2_ is also included as reference. (**b**) Raman spectra from undoped and Sn doped NiO samples acquired with a UV laser (λ = 325 nm).

XRD patterns from undoped and Sn doped samples are shown in Figure 2a. The results indicate that only for the NiO and NiO3 samples, all the diffraction peaks can be indexed with the characteristic NaCl structure of NiO (cubic structure, S.G. Fm-3m and lattice parameters a = b = c = 4.17 Å). However, upon a nominal atomic concentration of 6 at.%, weak contributions at 26.8°, 33.9° and 51.8° can be appreciated, increasing their relative intensity as a function of the Sn content. These peaks can be attributed to SnO_2_ rutile structure (S.G. P42/mnm and lattice parameters a = b = 4.74 Å and c = 3.18 Å), confirming the segregation of SnO_2_ upon a nominal atomic concentration of 6 at.%. Neither peaks from the precursors nor from other Sn-based or Ni-based oxides are observed in the XRD patterns.

The average crystallite dimensions (*D*) of NiO were calculated following Scherrer’s equation:

(1) $$D=\;\frac{K\lambda }{B\cos Ɵ}$$

where *K* is a dimensionless shape factor (0.9 in this work), *λ* is the X-ray wavelength (1.5404 Å), B is the line broadening at half the maximum intensity and Ɵ is the Bragg angle. Following Equation (1), the particle size was estimated for all of the samples, as shown in Table 1.

**Figure 3:**
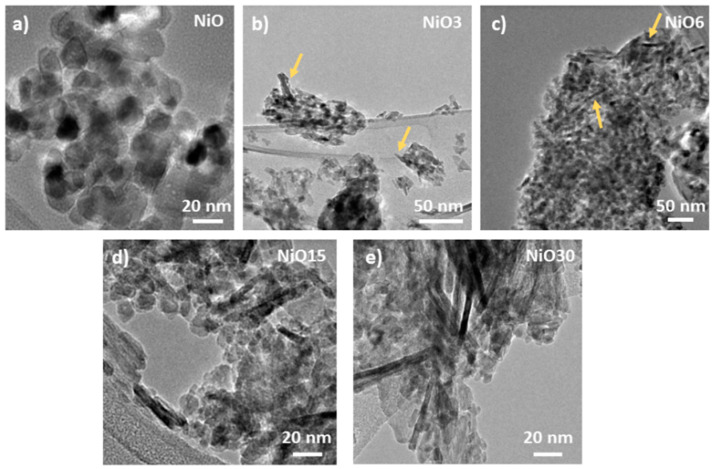
TEM images from (**a**) undoped NiO sample, (**b**) NiO3 sample, (**c**) NiO6 sample, (**d**) NiO15 sample and (**e**) NiO30 sample

The shape and intensity of the diffraction maxima indicate high crystallinity and nanometric character of the as-grown samples. In addition, as the Sn content increases, the average particle size decreases, as confirmed by the broadening of the diffraction peaks and the particle sizes estimated following Scherrer’s equation. The ionic radius of Ni^2+^ (0.69 Å) is similar to that of Sn^4+^ (0.69 Å), therefore the substitution of Ni^2+^ sites for Sn^4+^ is more favorable in the NiO lattice. According to the XRD results, Sn substitution in NiO reduces the crystallite size, as indicated by the broadening of the diffraction peaks shown in Figure 2a. This could be related to a different kinetic process during the synthesis since SnO_2_ and NiO exhibit different oxidation kinetics, as well as different reactivities. In general, the decrease in particle size is usually attributed to the presence of dopants that can modify the kinetics of the synthesis of nanostructures, as reported by several authors. For example, Wang et al. demonstrated that Sn prevents NiO nanocrystals from growing and combining into larger crystals [6]. Ponnusamy et al. reported a decrease in the particle size of NiO with Fe incorporation, due to the presence of Fe^3+^, which increases the nucleation points in the NiO nanoparticles [23]. In this work, the presence of Sn in the NiO lattice, and its possible incorporation in different coordination environments, could lead to a decrease in the particle size similar to those previously described, favoring the inhibition of the growth of NiO nanostructures. The Raman spectra of both pure NiO and Sn-doped NiO samples are shown in Figure 2b. The NiO Raman spectrum exhibits two broad peaks around 546 and 1059 cm^−1^, corresponding to one-phonon mode (LO) and two-phonon mode (2LO), respectively, of NiO [24,25,26]. LO mode is commonly attributed to Ni^2+^-O stretching mode [27], which is expected to be weak or not observed in the Raman signal from stoichiometric NiO. The presence of this LO mode in the Raman spectra from the analyzed nanoparticles could be related to the presence of defects in NiO, such as Ni vacancies [28,29] or the presence of Ni^3+^ [30]. The Raman spectra from Sn doped NiO nanostructures show additional weak modes centered on 702 and 875 cm^−1^ corresponding to 2TO and TO+LO optical modes in NiO [25,26], respectively, not showing Raman peaks corresponding to SnO_2_ or other Sn-based compounds. However, the main difference among these spectra is the blue-shift (higher than 20 cm^−1^ for LO mode) observed in the Raman peaks from Sn doped NiO, as compared with bare NiO. It is well known that a decrease in the particle size, similar to that observed in this work, could lead to a red-shift in the Raman spectra due to confinement effects and relaxation of the surface [31,32], as reported by diverse authors. However, an opposite behavior can be observed in this case since the decrease in the particle size leads to blue-shifted Raman peaks. Hence, apart from the low dimensions of the nanostructures, some other phenomena related to the doping process should be considered in the analysis [31]. Actually, Varshney et al. [33] observed a blue-shift related to the direct substitution of Ni^2+^ by Sn^4+^ and a decrease in the lattice parameters with Sn incorporation. In our case, there is not a clear trend between lattice parameters and Sn incorporation, therefore other factors, such as the presence of Ni vacancies, Ni^3+^ or the presence of Sn in different coordination environments, should be considered. EDS measurements shown in Supplementary Materials Figure S1 confirm the presence of Ni, Sn and O in the samples. The atomic concentrations of all the samples, estimated from the analysis of the corresponding EDS signal, are shown in Table S1. The amount of Sn varies from 0 to 14.4 at.% as a function of the initial Sn concentration employed during the synthesis. EDS mappings confirm the homogeneous distribution of Sn in the analyzed nanopowders. Theoretical and experimental atomic Sn concentrations are similar for samples with Sn content below 6%. However, upon this nominal concentration, a deviation of the experimental values can be observed when compared with theoretical ones. Hydrothermal synthesis usually requires an exhaustive control of the experimental conditions, such as temperature, reaction time or pH. Among these, pH control is an essential key to control the dopant concentration. Increasing pH value would lead to an increase of Sn concentration; however, impure samples would be obtained. This is because for pH values above 10, Sn^2+^ cations could suffer dismutation process, obtaining Sn^0^ and Sn^4+^. Of course, this Sn^0^ could be easily oxidized to Sn^2+^ or Sn^4+^ following thermal treatments, but at temperatures higher than 450 °C, SnO_2_ segregation is achieved as well as increased dimensions. Taking this into account, the control of experimental conditions allows us to obtain both Sn doped NiO nanostructures and NiO/SnO_2_ structures. An increase in the Sn concentration could be achieved with higher pH values; however, Sn doped NiO nanostructures would be more difficult to obtain. Figure S1b shows the compositional mapping from the NiO3 sample, while the corresponding EDS spectra for all samples are included in Figure S1c. The morphologies of the NiO nanostructures were deeply analyzed by TEM, as shown in Figure 3. For the undoped NiO sample, spherical nanoparticles with homogeneity in size and morphology can be observed. Averaged sizes around 12 nm were estimated for NiO nanoparticles from the TEM analysis (Figure 3a), in agreement with XRD results. It should be remarked that Sn incorporation promotes the formation of some elongated nanostructures in the form of nanosticks in the Sn doped samples (marked with arrows in Figure 3b,c), not observed in the undoped NiO. These elongated nanostructures exhibit lengths of tens of nm with widths of few nm. The amount and length of these nanosticks increase with the Sn content, as seen in Figure 3d,e. In the case of the NiO30 sample, these elongated structures reach 100 nm in length. The formation of nanoparticles with dimensions lower than for undoped NiO can be also observed in the Sn doped samples, because these nanoparticles are the predominant morphology for all samples. The decrease in particle size with Sn incorporation can be also appreciated in the TEM micrographs, in agreement with XRD results. In order to achieve a deeper understanding of the relationship between the Sn content and the variable morphology observed by TEM, HRTEM analysis was performed for the NiO sample, as a reference, and NiO30 samples, where the dimensions of the nanosticks allow us to perform a detailed study of their microstructure.

**Figure 4:**
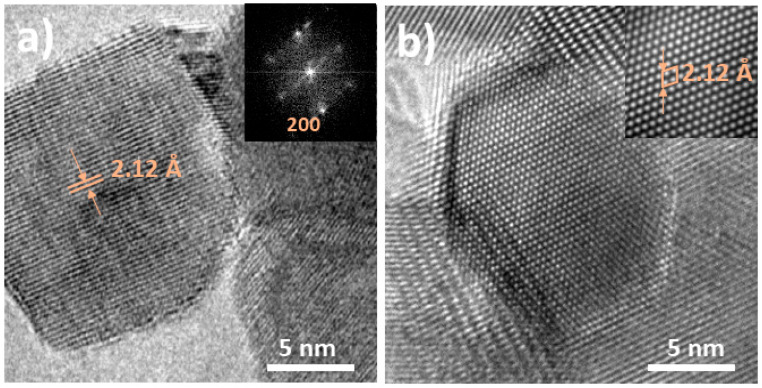
HRTEM images from undoped NiO sample. (**a**) Isolated NiO nanoparticle with the corresponding SAED pattern, and (**b**) NiO nanoparticle with atomic resolution and the corresponding I-FFT, confirming the NiO cubic structure.

**Figure 5:**
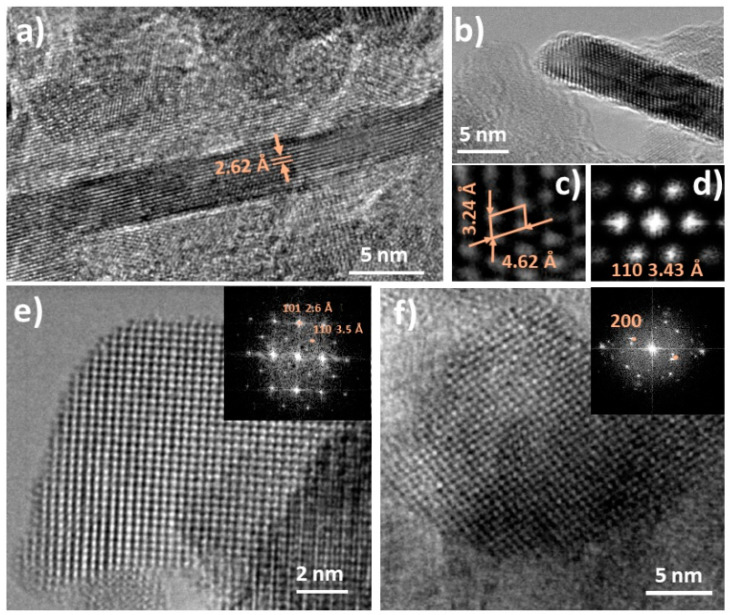
HRTEM images from the NiO30 sample. (**a**) Nanostick with interplanar distances corresponding to SnO_2_. (**b**) NiO30 nanostick with atomic resolution and their corresponding (**c**) I-FFT and (**d**) SAED pattern, confirming the SnO_2_ rutile structure. (**e**) SnO_2_ nanoparticle with the corresponding SAED pattern. (**f**) NiO nanoparticle with the corresponding SAED pattern.

Figure 4 shows the HRTEM analysis performed for the NiO sample. Figure 4a shows an isolated nanoparticle with dimensions around 10 nm, where interplanar distances of 2.12 Å can be measured, and corresponding to (200) plane of cubic NiO. The selected area electron diffraction (SAED) pattern included in the inset confirms the rock-salt structure of the analyzed NiO nanoparticle. Figure 4b shows another nanoparticle with dimensions around 7 nm, where the corresponding Inverse Fast Fourier Transform (I-FFT)(shown in the inset), shows the primitive cell of NiO with cubic structure. The NiO30 sample was analyzed following the same procedure. Figure 5 shows the HRTEM images from the NiO30 sample, with the highest concentration of Sn, where two different morphologies in form of nanoparticles and nanosticks were observed in the corresponding low magnification TEM images shown in Figure 3. Figure 5a shows a nanostick with dimensions greater than 20 nm in length and 2 nm in width. Interplanar distances of 2.62 Å can be measured in this case, corresponding to (101) plane of SnO_2_ with rutile structure. Figure 5b shows an HRTEM image from another nanostick with atomic resolution and the corresponding I-FFT (Figure 5c) and SAED pattern (Figure 5d), confirming that nanosticks are formed mainly by SnO_2_ with rutile structure. However, some of the nanosticks show defective regions and local interplanar distances slightly distorted from rutile SnO_2_. In addition, although in a lower concentration SnO_2_ nanoparticles can be also observed in this sample, as shown in Figure 5e, where the SAED pattern included shows the diffraction points corresponding to (101) and (110) planes of SnO_2_. Figure 5f shows a NiO nanoparticle with the respective SAED pattern shown in the insert, where (200) planes corresponding to NiO cubic structure can be indexed. These results suggest that the NiO30 sample contains two main morphologies: NiO nanoparticles and SnO_2_ nanosticks, although some SnO_2_ nanoparticles can also be observed in a low concentration. In order to confirm these results, local EDS spectra were acquired in different points of the NiO30 nanopowders.

Table S2 shows the atomic percent of Ni, Sn and O acquired in regions with high amount of nanoparticles or nanosticks. These values confirm that the regions with nanoparticles are Ni-rich while the regions with nanosticks are Sn-rich, therefore the formation of Ni doped SnO_2_ nanosticks and Sn doped NiO nanoparticles should also be considered in this work. The HRTEM analysis from the NiO15 sample shows similar features to those described for NiO30, as shown in Figure S2. In this case, two different nanosticks were analyzed showing interplanar distances around 2.6 Å, corresponding to (101) plane of rutile–SnO_2_ structure. Considering both XRD results and HRTEM analysis, the formation of nanosticks could also partially explain the further decrease in the particle size of NiO previously described, since the nanosticks formation does not contribute to the growth of larger NiO nanocrystals. Based on our previous work, the synthesis of SnO_2_ following a similar hydrothermal method favors the formation of homogeneous rounded nanoparticles [22]. In this work, the presence of both Ni and Sn favors the formation of nanosticks; therefore, Ni and Sn could act as catalysts promoting the formation of elongated SnO_2_ and Ni doped SnO_2_ nanosticks.

**Figure 6:**
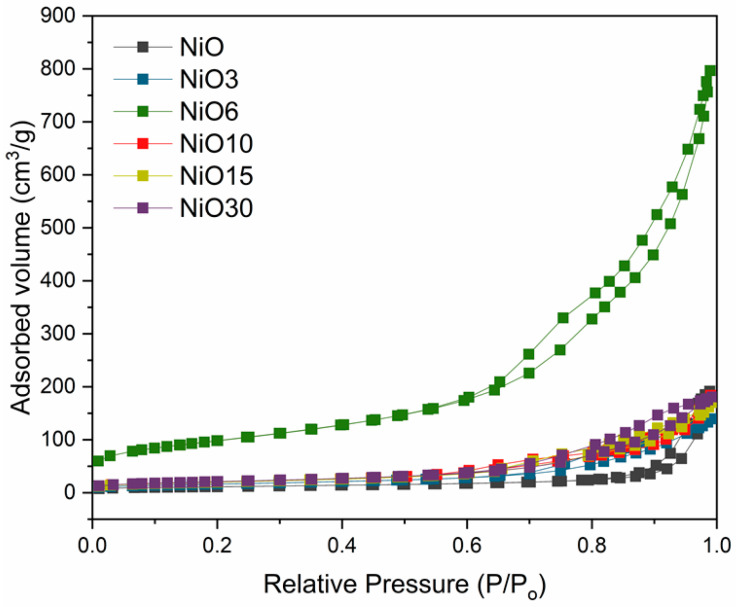
Nitrogen adsorption–desorption isotherms from undoped and Sn doped samples.

The surface area of the undoped and Sn doped samples were obtained using the nitrogen adsorption–desorption isotherms, showing the corresponding curves in Figure 6. It can be seen that all of the samples display a characteristic hysteresis loop indicating the existence of porous structures. The BET surface areas of all the samples are listed in Table 1. It is noticeable that in a range of nominal atomic concentrations between 0 < x ≤ 6, the surface area gradually increases, achieving remarkable values of 342 m^2^/g for the NiO6 sample, which is consistent with the decrease of particle size observed by XRD, which contributes to the increase of the surface area. However, upon a nominal atomic concentration of 6%, a drastic decrease in the surface area can be observed. It should be noted that upon this concentration, the segregation of SnO_2_ was observed, providing the formation of nanosticks in a larger concentration and with higher dimensions. This fact could lead to a decrease of the number of free positions where the adsorbate can be adsorbed, thereby decreasing the value of the surface area. The most remarkable feature of these results is the high surface area observed for the NiO6 sample, the one in which SnO_2_ starts to segregate. Different values can be found in the literature for the surface area of Sn doped NiO, based on the particle size or morphology. For example, Wang et al. [6] reported values around 108 m^2^/g for Sn-doped three-dimensionally ordered macroporous NiO with a particle size similar to that observed for the NiO6 sample. Gu et al. [34] reported different values of surface area as a function of the Sn doped NiO morphology: 61.5 m^2^/g for Sn doped NiO microspheres and 38.1 m^2^/g for microcubes. Kim et al. [14] reported a value of 87.9 m^2^/g for Sn doped NiO microspheres. In this work, the value of the surface area for the NiO6 sample is clearly higher than previously reported values, which makes this material a promising candidate for applications where surface area plays a key role, such as gas sensors, photocatalysis or supercapacitors. XRD and EDS results point to a solubility limit of Sn in NiO below 4.7 at.%, as SnO_2_ starts to segregate in the NiO6 sample, which also exhibits high surface area values and an initiated growth on nanosticks, confirmed by BET and TEM measurements.

**Figure 7:**
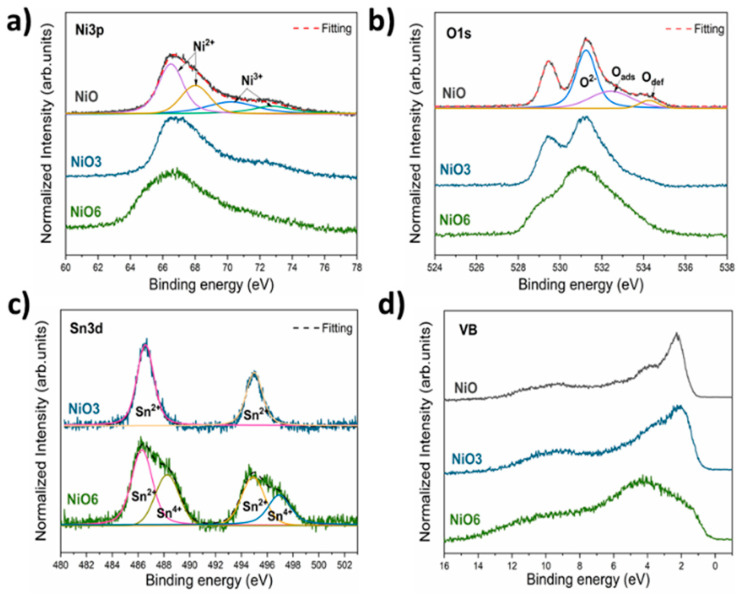
XPS spectra from (**a**) Ni 3p, (**b**) O 1s, (**c**) Sn 3d core levels and (**d**) valence band region acquired for the NiO, NiO3 and NiO6 samples.

Hence, in order to achieve a deeper understanding of the Sn doping NiO process, surface sensitive XPS measurements were performed for the samples just below (NiO3) and over (NiO6), the solubility limit of Sn in NiO. XPS spectra of the NiO sample were also acquired as a reference. The XPS spectra were calibrated using the C1s signal from adventitious carbon and deconvolutions, which were carried out using Voigt functions after an appropriate Shirley background correction. Ni 3p, O 1s, Sn 3d core levels and valence band region were acquired, as shown in Figure 7. Figure 7a shows the XPS spectra from Ni 3p core levels, where four bands can be observed. The low energy bands at 66.4 and 68.1 eV correspond to Ni 3p_3/2_ and Ni 3p_1/2_, respectively, due to Ni^2+^ in NiO [35,36], whereas the high energy bands at 70.1 and 72.4 eV are related to Ni 3p_3/2_ and Ni 3p_1/2_ from Ni^3+^. These results confirm the presence of a variable concentration of Ni^3+^ in the probed samples, confirming the nonstoichiometry found in the Raman spectra of the samples. Nonstoichiometric NiO contains Ni vacancies, and some Ni^2+^ is oxidized to Ni^3+^ in order to maintain charge neutrality [28,30]. The Ni^3+^/Ni^2+^ ratio, estimated from XPS spectra, indicates values around 0.42 for the NiO and NiO3 samples and 0.22 for the NiO6 sample, which indicates a lower presence of Ni^3+^ as the concentration of Sn rises.

Figure 7b shows XPS spectra from O 1s core level. It should be noted that the contribution around 529 eV observed with a higher relative intensity for the NiO and NiO3 samples corresponds to C 1s from the sample holder, therefore this signal will not be considered. The spectra from all the samples are dominated by a contribution centered at 531.2 eV, which corresponds to lattice O^2−^ in NiO [1,36]. Two contributions at higher energies (532.4 and 543.3 eV) can be distinguished for all the samples, increasing their relative intensity for the NiO6 sample with higher Sn content. These two contributions are usually attributed to adsorbed oxygen species and defect oxygen [1,6,36], respectively.

Sn 3d core levels from NiO3 and NiO6 are shown in Figure 7c, which confirm the presence of Sn in these samples. For the NiO3 sample, the Sn 3d spectrum is formed by two peaks centered at 486.5 and 495 eV, corresponding to Sn 3d_5/2_ and Sn 3d_3/2_, respectively [36,37]. In the case of the NiO6 sample, two additional contributions with lower relative intensity centered at 488 and 496.5 eV can also be observed. Therefore, it can be confirmed that the lower energy contributions correspond to Sn^2+^ while the higher energy contributions, observed only for the NiO6 sample, correspond to Sn^4+^. The presence of Sn with different oxidation states leads to changes in the Ni^3+^/Ni^2+^ ratio, as previously discussed. It is well known that substitutional Sn^4+^ usually promotes the formation of defects (oxygen defects or Ni vacancies) in order to maintain the charge neutrality of the system. Two different charge compensation mechanisms [6,38] (electronic compensation or ionic compensation) have been previously reported for doped NiO. On the one hand, when Ni^2+^ is replaced by isovalent Sn^2+^, Ni vacancies should not be altered. This fact can be confirmed by considering the similar values estimated for Ni^3+^/Ni^2+^ ratios for the NiO and NiO3 samples. However, for the NiO6 sample, where Sn^4+^ was also detected, a decrease in the Ni^3+^/Ni^2+^ ratio can be observed, leading to a lower concentration of Ni vacancies. It should be noted that for this sample, an increase in the relative intensity of adsorbed oxygen species and defective oxygen was observed, which could alter the concentration of Ni vacancies. Therefore, a combination of electronic and ionic compensation mechanisms should be considered in this work. Valence band region was also analysed, as shown in Figure 7d. The XPS spectra from the NiO and NiO3 samples are dominated by a contribution around 2 eV attributed to Ni 3d states, [39] whereas the spectrum from the NiO6 sample is dominated by a contribution around 4 eV related to O 2p states [39]. These results agree with the values of Ni^3+^/Ni^2+^ ratios previously estimated, and with the relative intensity of oxygen species shown in Figure 7b.

In addition, the difference between the Fermi level and the maximum of the valence band (E_F_–E_VBM_) was also estimated with values around 1.5, 1.1 and 0.6 eV for the NiO, NiO3 and NiO6 samples, respectively. These results indicate a higher p-type character for the Sn doped samples. The combination of XRD, BET and XPS results reveal a decrease in the particle size, an increase of the surface area and a higher p-type character with Sn incorporation, confirming that Sn doping can be a successful tool for the improvement of several physical and chemical properties.

**Figure 8:**
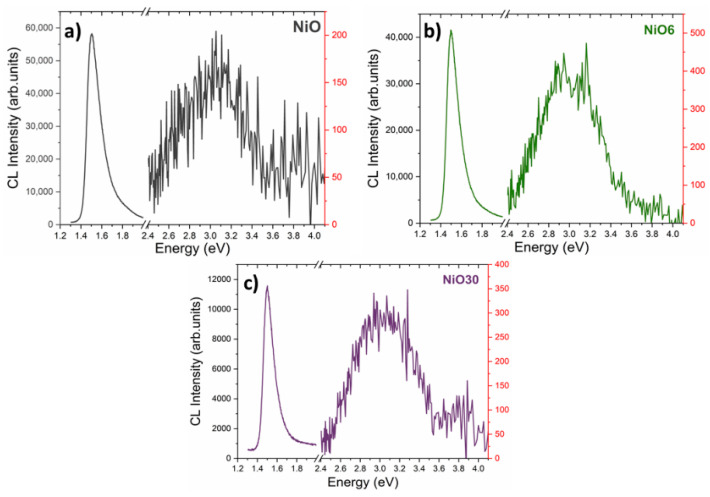
Cathodoluminescence (CL) spectra acquired at room temperature from (**a**) NiO, (**b**) NiO6 and (**c**) NiO30 samples.

Finally, in order to study the influence of Sn doping and/or SnO_2_ segregation in the luminescent properties of NiO, CL spectra were acquired for the samples with the lowest (NiO) and highest (NiO30) amount of Sn, and for the NiO6 sample, which demonstrated a particular behavior. Figure 8a–c shows CL spectra for NiO, NiO6 and NiO30, respectively, acquired at room temperature. For all the samples, CL signal is dominated by two different contributions in the near IR region and UV, centered at 1.5 and 3.1 eV, respectively. The contribution centered at 1.5 eV, which relative intensity decreases as a function of Sn content, is related to Ni^2+^ defect states [40], as previously reported. The decrease in the relative intensity of these near-IR emissions could be related with the lower Ni^2+^ defect states owing to the substitution of Ni^2+^ by Sn^4+^, as confirmed by XRD and XPS measurements. The contribution centered at 3.1 eV shows a lower intensity and is usually attributed to self-trapped d-d charge transfer excitons formed by coupled Jahn–Teller Ni^2+^ and Ni^3+^ centers [41,42,43], although their origin is still unclear. The presence of cations with different oxidation states, as confirmed by XPS results, directly affect the defects structure, and therefore to the electronic and optical properties.

## 4. Conclusions

In this work, positive aspects regarding to the potential applicability of the Sn doped NiO nanostructures in supercapacitors, sensors or photocatalysis are highlighted. In summary, a series of undoped and Sn doped NiO nanostructures were synthesized by an optimized hydrothermal method. The XRD results indicate that upon a 4.7 at.% of Sn, the segregation of SnO_2_ in NiO is promoted. TEM and HRTEM analysis confirm that Sn and Ni can act as catalysts, leading to the growth of SnO_2_ nanosticks with dimensions of tens of nm length and a few nm width. The size of these nanosticks increases as the amount of Sn rises in the samples. Both NiO and SnO_2_ nanostructures are formed during the synthesis, leading to p-n heterojunctions. High values of surface area are estimated, mainly for the NiO6 sample (342.4 m^2^/g). Sn doping is confirmed for the NiO nanoparticles, while SnO_2_ nanosticks could also be Ni doped. The two possible charge compensations mechanisms induced by the presence of Sn with different oxidation states provide a new strategy to increase the p-type character of NiO. Finally, the defects structure of NiO (mainly Ni vacancies) play a key role in the luminescence properties as observed by CL measurements.

## Figures and Tables

**Table 1:** Structural parameters, particle size and surface area from Sn doped and undoped NiO samples.

Sample	Lattice Parameters ^a^ a = b = c (Å)	D_NiO_^a^ (nm)	D_NiO_^b^ (nm)	Surface Area ^c^ (m^2^/g)
NiO	4.178	10.6	12	39.4
NiO3	4.181	7.9	8.1	56.8
NiO6	4.180	7.3	7.6	342.4
NiO10	4.183	6.9	6.6	71.7
NiO15	4.181	6.8	6.2	71.2
NiO30	4.167	5.7	5.4	72.6

^a^ Determined via XRD; ^b^ Determined via TEM; ^c^ Determined via BET.

## Supplementary Materials

The following are available online at https://www.mdpi.com/2079-4991/11/2/444/s1, Figure S1: SEM image and compositional mappings from NiO3 samples, and EDS spectra from all analyzed samples. Figure S2: HRTEM analysis from NiO15 sample. Table S1: Atomic concentration of O, Ni and Sn from undoped and Sn doped samples acquired by EDS. Table S2: Atomic percent of O, Ni and Sn acquired from NiO30 sample.
