# Supplementary material for: Towards Control of the Size, Composition and Surface Area of NiO Nanostructures by Sn Doping
Source: Nanomaterials (Basel). 2021 Feb 10;11(2):444. doi: 10.3390/nano11020444 (PMC7916375; doi:10.3390/nano11020444)
Supplement: Supplementary file 1 [file nanomaterials-11-00444-s001.pdf]

nanomaterials

MDPI

Supplementary Materials

Towards Control of the Size, Composition and Surface Area of NiO Nanostructures by Sn Doping

María Taeño [1,*], David Maestre [1], Julio Ramírez-Castellanos [2], Shaohui Li [3], Pooi See Lee [3] and Ana Cremades [1]

[1] Departamento de Física de Materiales, Facultad de Ciencias Físicas, Universidad Complutense de Madrid, 28040 Madrid, Spain; davidmaestre@fis.ucm.es (D.M.); cremades@fis.ucm.es (A.C.)
[2] Departamento de Química Inorgánica, Facultad de Ciencias Químicas, Universidad Complutense de Madrid, 28040 Madrid, Spain; jrcastel@quim.ucm.es
[3] School of Materials Science and Engineering, Nanyang Technological University, 50 Nanyang Avenue, 639798 Singapore, Singapore; lishaohui@ntu.edu.sg (S.L.); pslee@ntu.edu.sg (P.S.L.)
* Correspondence: m.taeno@ucm.es

EDS measurements were carried out in order to determine the Sn concentration in the doped samples as shown in Table S1. Sn content increases from Ni03 to Ni030, with values ranging from 2.15 to 14.4 at.%.

Table S1. Atomic concentration of O, Ni and Sn from undoped and Sn doped NiO samples acquired by EDS.

	Atomic %		
**Sample**	**O**	**Ni**	**Sn**
NiO	46.5 ± 2.6	53.4 ± 2.5	-
NiO3	44.6 ± 2.1	53.3 ± 2.3	2.15 ± 0.2
NiO6	45.9 ± 1.9	49.4 ± 2.1	4.71 ± 0.4
NiO10	35.5 ± 1.4	57.6 ± 2.1	6.78 ± 0.5
NiO15	42.3 ± 1.8	48.7 ± 1.7	9.12 ± 0.7
NiO30	51.7 ± 2.4	34.3 ± 1.3	14.4 ± 1.1

Figure S1a shows a SEM image from NiO3 sample where agglomerated nanoparticles can be observed. In addition, compositional mappings have been acquiered with Ni, O and Sn signal, confirming a homogeneus distribution of Sn as shown in Figure S1b. EDS spectra from all the samples are shown in Figure S1c, where only Ni, Sn and O can be detected, within the limit of the technique.

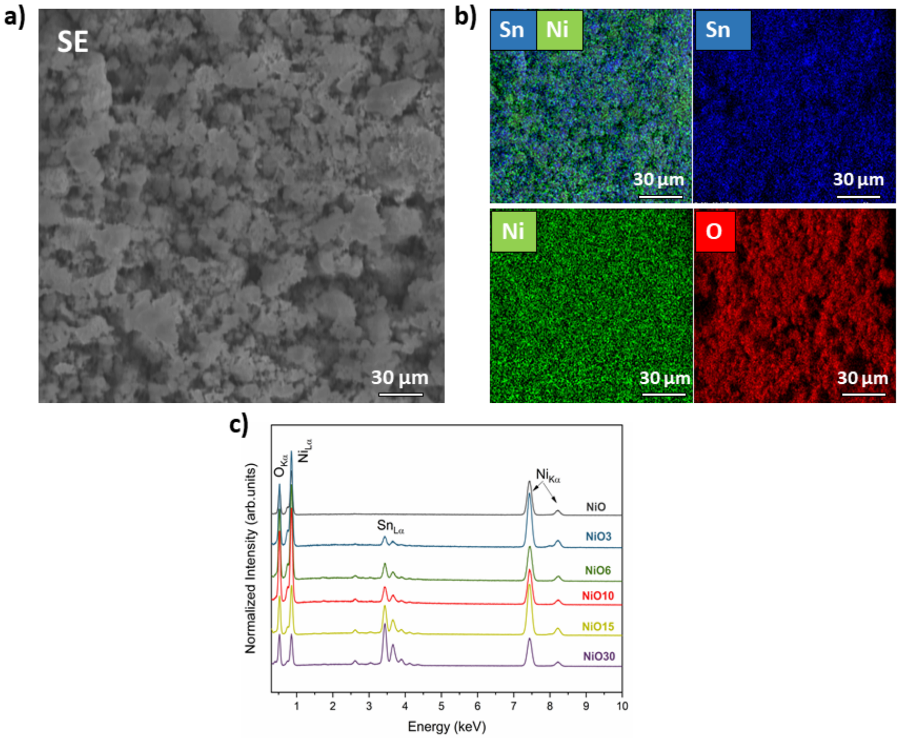

Figure S1. (**a**) SEM image from NiO3 sample with the corresponding (**b**) compositional mappings acquired with Sn, Ni and O signal, and (**c**) the corresponding EDS spectra from undoped and Sn doped samples.

In order to confirm the composition of the different morphologies observed in the HRTEM images (Figures 3 and 4), punctual quantifications were performed in different regions with high amount of nanosticks or nanoparticles, as shown in Table S2. These results confirm that the nanosticks are Sn-rich whereas nanoparticles Ni-rich.

Table S2. Atomic percent of O, Ni and Sn acquired in regions with nanoparticles and regions with nanosticks from NiO30 sample.

	**Atomic %**		
**Analyzed region**	**O**	**Ni**	Sn
Nanoparticles	49.8	35.1	15.1
Nanosticks	55.3	15.1	27.6

HRTEM analysis has been performed for NiO15 sample where the segregation of SnO_2 was observed by XRD. Figure. S2a shows two nanosticks where interplanar distances around 2.6–2.7 Å can be measured, as well as the corresponding SAED pattern (shown in the inset). Both results confirm the presence of rutile SnO_2. Figure. S2b shows another nanostick with atomic resolution with the corresponding I-FFT (Figure. S2c), where lattice parameters of SnO_2 can be measured and the corresponding SAED pattern (Figure. S2d).

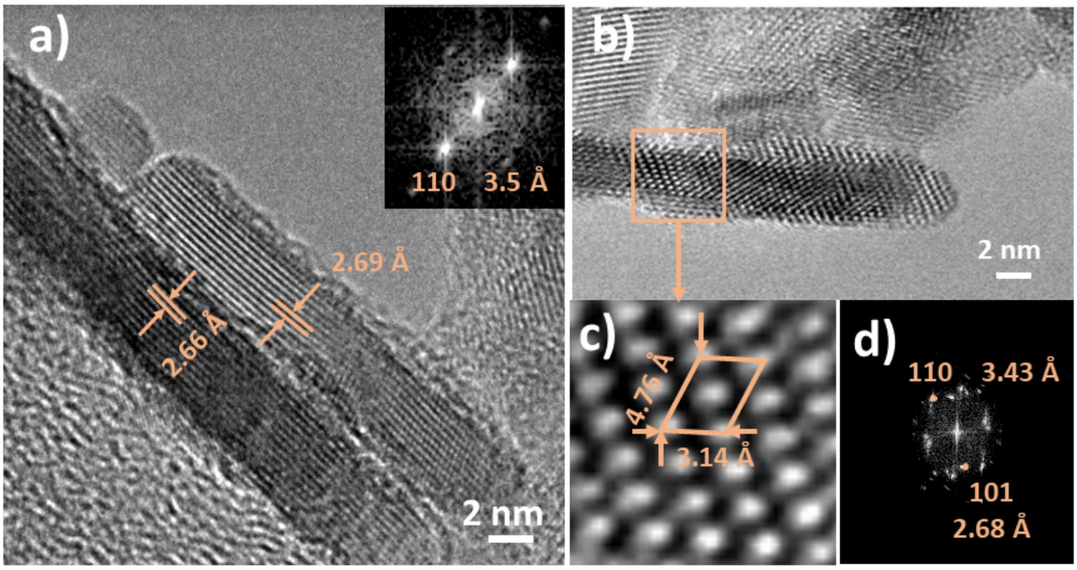

Figure S2. HRTEM images from NiO15 sample. (**a**) Nanostick with interplanar distances corresponding to SnO_2 and the corresponding SAED pattern. (**b**) NiO15 nanostick with atomic resolution and their corresponding (**c**) I-FFT and d) SAED pattern confirming the SnO_2 rutile structure.
